# Performance of Electronic Prediction Rules for Prevalent Delirium at Hospital Admission

**DOI:** 10.1001/jamanetworkopen.2018.1405

**Published:** 2018-08-24

**Authors:** Christopher W. Halladay, Andrea Yevchak Sillner, James L. Rudolph

**Affiliations:** 1 Center of Innovation in Long Term Services and Supports, Providence Veterans Affairs Medical Center, Providence, Rhode Island; 2 College of Nursing, The Pennsylvania State University, University Park; 3 Brown University, Warren Alpert Medical School and School of Public Health, Providence, Rhode Island

## Abstract

**Importance:**

Delirium at admission is associated with increased hospital morbidity and mortality, but it may be missed in up to 70% of cases. Use of a predictive algorithm in an electronic medical record (EMR) system could provide critical information to target assessment of those with delirium at admission.

**Objectives:**

To develop and assess a prediction rule for delirium using 2 populations of veterans and compare this rule with previously confirmed rules.

**Design, Setting, and Participants:**

In a diagnostic study, randomly selected EMRs of hospitalized veterans from the Veterans Affairs (VA) External Peer Review Program at 118 VA medical centers with inpatient facilities were reviewed for delirium risk factors associated with the National Institute for Health and Clinical Excellence (NICE) delirium rule in a derivation cohort (October 1, 2012, to September 30, 2013) and a confirmation cohort (October 1, 2013, to March 31, 2014). Delirium within 24 hours of admission was identified using key word terms. A total of 39 377 veterans 65 years or older who were admitted to a VA medical center for congestive heart failure, acute coronary syndrome, community-acquired pneumonia, and chronic obstructive pulmonary disease were included in the study.

**Exposure:**

The EMR calculated delirium risk.

**Main Outcomes and Measures:**

Delirium at admission as identified by trained nurse reviewers was the main outcome measure. Random forest methods were used to identify accurate risk factors for prevalent delirium. A prediction rule for prevalent delirium was developed, and its diagnostic accuracy was tested in the confirmation cohort. This consolidated NICE rule was compared with previously confirmed scoring algorithms (electronic NICE and Pendlebury NICE).

**Results:**

A total of 27 625 patients were included in the derivation cohort (28 118 [92.2%] male; mean [SD] age, 75.95 [8.61] years) and 11 752 in the confirmation cohort (11 536 [98.2%] male; mean [SD] age, 75.43 [8.55] years). Delirium at admission was identified in 2343 patients (8.5%) in the derivation cohort and 882 patients (7.0%) in the confirmation cohort. Modeling techniques identified cognitive impairment, infection, sodium level, and age of 80 years or older as the dominant risk factors. The consolidated NICE rule (area under the receiver operating characteristic [AUROC] curve, 0.91; 95% CI, 0.91-0.92; *P* < .001) had significantly higher discriminatory function than the eNICE rule (AUROC curve, 0.81; 95% CI, 0.80-0.82; *P* < .001) or Pendlebury NICE rule (AUROC curve, 0.87; 95% CI, 0.86-0.88; *P* < .001). These findings were confirmed in the confirmation cohort.

**Conclusions and Relevance:**

This analysis identified preexisting cognitive impairment, infection, sodium level, and age of 80 years or older as delirium screening targets. Use of this algorithm in an EMR system could direct clinical assessment efforts to patients with delirium at admission.

## Key Points

**Question:**

What are the most influential factors associated with prevalent delirium at admission for medical patients?

**Findings:**

In this diagnostic study of electronic medical records for 39 377 veterans, cognitive impairment, infection, sodium level, and age of 80 years or older were the most dominant factors associated with delirium at admission. Use of these factors was an improvement over previously confirmed prediction rules.

**Meaning:**

Use of this algorithm in an electronic medical record system may help to identify patients with delirium within 24 hours of hospital admission for clinical evaluation and appropriate intervention after additional prospective evaluation.

## Teaser

This diagnostic study of hospitalized veterans uses the UK National Institute for Health and Clinical Excellence (NICE) framework and delirium clinical practice guidelines to compare the performance of prediction rules for prevalent delirium among older adults at hospital admission.

## Introduction

Delirium is a short-term change in attention and awareness that typically affects hospitalized, high-risk older adults.^1^ Many older adults who present to the emergency department and are subsequently admitted to the hospital have prevalent delirium. Han et al^2^ found that up to 15% of patients present to the emergency department with delirium, and delirium at acute care admission for older adults has a reported prevalence of 18% to 39%.^3,4,5^ This prevalence increases to 57% among older adults admitted with a diagnosis of preexisting dementia.^6^

Despite the high prevalence, delirium is poorly recognized in the acute care setting. Early research in delirium found that the diagnosis was missed in up to two-thirds of older adults,^7^ and despite advances in delirium science, delirium continues to go unrecognized at presentation to the emergency department or hospital admission.^8,9,10^ Although prevalent delirium cannot be prevented, failure to recognize delirium is associated with increased length of stay, health complications, discharge to a skilled nursing facility,^4^ and increased risk of death.^11,12,13^ In addition, delirium is associated with increased distress for individuals experiencing delirium and their families, as well as health care professionals.^14,15^

Delirium prediction algorithms have been used to stratify those at highest risk for delirium so that increased resources and efforts can be allocated to those in greatest need.^16,17,18^ Identification of those at highest risk for delirium allows for improved clinical efficiency and more timely recognition and diagnosis of delirium. Older adults admitted to the hospital with delirium need diagnosis, identification of underlying risk factors and potential causes, and appropriate management of and intervention for delirium.^19^ The National Institute for Health and Clinical Excellence (NICE) in the United Kingdom performed a comprehensive systematic review and meta-analysis of delirium risk factors as part of a delirium clinical practice guideline.^20,21^ The factors identified in the NICE meta-analysis have been prospectively confirmed for delirium that develops after admission.^16,17^

The primary purposes of this study were to use the framework of the NICE meta-analysis and delirium clinical practice guidelines^21^ to compare the performance of 3 prediction rules for prevalent delirium and to consolidate the prediction rule components to the minimum information necessary to maximize predictive ability. We hypothesized that a consolidated prediction rule for delirium at admission would perform better than existing NICE-based delirium prediction rules. Having a singular, consolidated delirium prediction rule could target clinical efforts of screening toward those who need immediate evaluation for and diagnosis of delirium.

## Methods

### Sample

The sample for this analysis was drawn from the Veteran Affairs (VA) External Peer Review Program (EPRP) at 118 VA medical centers with inpatient facilities.^22^ Medical records of patients admitted for congestive heart failure, acute coronary syndrome, community-acquired pneumonia, and chronic obstructive pulmonary disease were randomly selected for electronic medical record (EMR) review by trained nurses for the presence of delirium and its risk factors. Interrater reliability assessments were built into the data collection process. From October 1, 2012, to September 30, 2013, a total of 27 625 VA hospital admissions were abstracted for patients 65 years or older; this group composed the derivation cohort. The confirmation cohort consisted of 11 752 patients from the EPRP sample from October 1, 2013, until March 31, 2014. This analysis follows the recommendations of the Transparent Reporting of a Multivariable Prediction Model for Individual Prognosis or Diagnosis (TRIPOD) reporting guideline (eAppendix in the Supplement).^23^ Data were not identifiable; however, because of the linear age variable, some veterans older than 90 years were identifiable. The VA Providence Institutional Review Board approved this analysis. This was a quality improvement project, and a waiver of informed consent from study participants was granted by the VA Providence Institutional Review Board and from the Health Insurance Portability and Accountability Act of 1996.

### NICE Delirium Prediction Rule

A systematic review and meta-analysis of delirium risk factors conducted by the NICE delirium clinical practice guideline identified 6 risk factors for delirium: (1) age with cutoff points at 65 and 80 years, (2) cognitive impairment, (3) illness severity, (4) infection, (5) fracture, and (6) visual impairment. These factors have been independently confirmed for incident and any delirium.^16^

### Electronic NICE Delirium Prediction Rule

The electronic NICE (eNICE) delirium prediction rule used criteria defined by the initial meta-analysis presented by the NICE delirium clinical practice guideline. All criteria were pulled from the EMR to confirm retrospective and prospective cases of delirium.^16^ Patients were admitted for general medical and surgical reasons, such as cardiac issues and infection. Age points were defined as 65 years and older and 80 years and older. Cognitive impairment was defined as EMR diagnosis or medication used to treat dementia at admission. Severity of illness was based on laboratory and vital sign data. Admitting diagnoses were screened to assess for infection and/or fracture. Visual impairment was determined by the EMR data. Delirium was assessed daily by a trained physician using *Diagnostic and Statistical Manual of Mental Disorders, Fourth Edition, Text Revision* criteria and was defined as any delirium at admission or during hospitalization.^16^

### Pendlebury NICE Delirium Prediction Rule

Pendlebury modified the NICE factors to confirm a rule for patients admitted to acute care hospitals.^17^ This was a prospective report, and delirium was screened for at admission and daily during hospitalization according to *Diagnostic and Statistical Manual of Mental Disorders, Fourth Edition* criteria by a physician. Criteria included in the prediction rule included cognitive impairment, age of 80 years and older, infection, visual impairment, and systemic inflammatory response syndrome (SIRS).^17^ Patients were admitted to the same medical team. Cognitive impairment was assessed using an abbreviated mental test score or Mini-Mental State Examination score and/or dementia diagnosis in the EMR. Visual impairment was noted in the EMR or if signs were evident during the patient visit. The Pendlebury NICE delirium prediction rule used SIRS criteria of respiratory rate, pulse, and white blood cell count as the criteria for acute illness,^17^ and these were taken from the EMR at admission.

### Proposed Consolidated NICE Delirium Prediction Rule

For the proposed consolidated NICE delirium prediction rule, age was abstracted from the medical record as the age at hospital admission and categorized as 65 years and older and 80 years and older. Cognitive impairment was defined as prior diagnosis of dementia in the EMR or outpatient prescription of a medication for dementia at admission (eg, donepezil). Severity of illness was calculated using an acute physiologic score from laboratory (ie, sodium level, bilirubin level, creatinine concentration, hematocrit, albumin level, blood urea nitrogen level, glucose level, and white blood cell count) and vital sign (pulse, respiratory rate, and blood pressure) data collected from the EMR with cutoffs similar to those of prior severity rules.^24^ Laboratory data used for the analysis were those most proximal to the admission to allow for the capture of laboratory values that were obtained in the emergency department. Infection and fracture were considered to be present if they were listed in the admission diagnoses. Infection data were abstracted by trained nurse reviewers and included the top 10 infections at the VA (ie, pneumonia, influenza, urinary tract infection, septicemia or sepsis, cellulitis, diverticulitis, peritonitis, appendicitis, osteomyelitis, and meningitis). Fracture was limited to the short-term presence of a femoral, vertebral, humeral, tibial, fibular, radial, or ulnar fracture. Visual impairment was based on review of the problem list and nursing admission notes for evidence of prior visual deficit diagnosis or for inability to correct vision during acute care admission. Multiple imputation was used for missing data (eTable 1 in the Supplement).

### Outcomes

The primary outcome for the consolidated NICE prediction rule was prevalent delirium, which was defined as the presence of 1 or more of the following terms or symptoms of delirium in the EMR within 24 hours of admission: (1) *delirium*, (2) *change in mental status*, (3) *disoriented*, (4) *confused*, (5) *unarousable*, (6) *lethargic*, and (7) *obtunded*. Interrater reliability, performed routinely within the EPRP, found 92% agreement among reviewers for prevalent delirium.^16^

### Statistical Analysis

The derivation and confirmation cohorts were compared using standardized differences. From the derivation cohort, we developed the eNICE and Pendlebury NICE prediction rules using the criteria outlined earlier from the initial studies.^16,17^

### Random Forest Modeling

The random forest algorithm was used to create the consolidated NICE score. Random forests are a classification tool that automatically constructs and classifies multiple decision trees and uses ensemble learning superimposed on regression to select independent variables. Random forest models reduce the model overfitting common with standard regression modeling^25^ by bootstrapping the decision trees consisting of the NICE predictive factors. The automated random forest algorithm provided an importance measure: mean decrease in accuracy (percentage increase in mean squared error; higher is better). On the basis of this measure, variable selection focused on net accuracy. More in-depth definitions of these importance measures can be found elsewhere.^25,26,27^ For our consolidated NICE model, we included age as a continuous variable to create an age cutoff using a separate random forest analysis. The same methods were applied to all other continuous variables, including laboratory measures, to find cutoffs. Because of the large size of our sample, we chose to eliminate the use of more complicated laboratory and vital scores (Acute Physiologic Assessment and Chronic Health Evaluation and SIRS criteria) and included each of the factors in the APACHE and SIRS criteria as individual features in our random forest model to determine the most important features when identifying prevalent delirium. All laboratory values were continuous in the data set to allow the random forest mechanism to capture the maximal possible information, after which we created an additive clinical diagnostic score based on the remaining important features. The feature weights were determined using random forest importance level. Clinical cutoff scores were created after the important features were selected using the modeling techniques. After producing a receiver operating characteristic (ROC) curve, we determined low-, intermediate-, and high-risk cut points for the consolidated NICE score, which were created for presentation.

We examined discriminatory performance of the 3 delirium prediction rules for delirium at admission in the derivation and confirmation cohorts using area under the ROC (AUROC) curve (*C* statistic) to test for model consistency and equality. Histograms and box plots were used to give a visual representation of the association between NICE score and delirium status in the derivation cohort. A density histogram provides a probability breakdown of each NICE score by delirium status. Comparison between the rules used a χ^2^ test. For comparisons, we set statistical significance at *P* < .05. Stata statistical software, version 14.2 (StataCorp) was used for data manipulation, eNICE score generation, and table creation. R, version 3.3.2 (R Foundation for Statistical Computing) was used to construct ROC curves. The randomForest program within R, version 3.3.2 was used to create the random forest algorithms.^27^

## Results

A total of 27 625 patients were included in the derivation cohort (28 118 [92.2%] male; mean [SD] age, 75.95 [8.61] years) and 11 752 in the confirmation cohort (11 536 [98.2%] male; mean [SD] age, 75.43 [8.55] years). Delirium at admission was identified in 2343 patients (8.5%) in the derivation cohort and 882 patients (7.0%) in the confirmation cohort. The derivation and confirmation cohorts are compared in Table 1. Although statistically significant differences were found between the derivation and confirmation cohorts, the actual differences were small and not clinically significant. For example, the analysis identified a difference between hematocrit in the derivation vs confirmation cohort (35.3% vs 34.9% [to convert to a proportion of 1.0, multiply by 0.01]; *P* < .001), which is statistically but not clinically significant. Means and percentages of vital signs, medications, laboratory values, and NICE scores across both cohorts were clinically similar but often statistically different.

**Table 1.** Characteristics of the Derivation and Confirmation Cohorts^a^

Characteristic	Derivation Cohort (n = 27 625)	Confirmation Cohort (n = 11 752)	Standardized Difference
Age, mean (SD), y	75.95 (8.61)	75.43 (8.55)	−0.007
Age ≥80 y	9953 (36.0)	3985 (33.9)	−0.044
Male	27 118 (98.2)	11 536 (98.2)	0
Fracture	351 (1.3)	142 (1.2)	−0.006
Infection	9180 (33.2)	3699 (31.5)	−0.038
Dementia diagnosis	3356 (12.1)	1290 (11.0)	−0.037
Dementia medications	950 (3.4)	338 (2.9)	−0.032
Visual impairment	12 667 (45.9)	5051 (43.0)	−0.0579
Pulse, mean (SD), beats/min	80.94 (18.12)	80.12 (16.64)	−0.003
Respiratory rate, mean (SD), breaths/min	19.35 (3.51)	19.17 (3.21)	−0.016
Systolic blood pressure, mean (SD), mm Hg	134.03 (23.97)	134.98 (22.81)	0.002
Diastolic blood pressure, mean (SD), mm Hg	73.74 (13.27)	74.47 (12.99)	0.004
Arterial pressure, mean (SD), mg/dL	93.61 (14.80)	94.41 (14.39)	0.004
Creatinine level, mean (SD), mg/dL	1.42 (1.24)	1.43 (1.51)	0.005
Blood urea nitrogen level, mean (SD), mg/dL	25.88 (16.53)	25.69 (16.56)	−0.001
Glucose level, mean (SD), g/dL	136.28 (58.79)	135.00 (56.41)	−0.022
Albumin level, mean (SD), mg/dL	3.31 (0.60)	3.29 (0.59)	−0.057
Bilirubin level, mean (SD), mg/dL	0.87 (0.97)	0.84 (0.62)	−0.043
White blood cell count, mean (SD), /μL	9690 (5700)	9650 (5510)	−0.001
Hematocrit, mean (SD), %	35.27 (5.91)	34.90 (5.94)	−0.011
eNICE score, mean (SD)	5.11 (2.79)	4.90 (2.71)	−0.028
Pendlebury NICE score, mean (SD)	2.24 (1.73)	2.08 (1.67)	−0.056
Consolidated NICE score, mean (SD)	1.45 (1.59)	1.33 (1.51)	−0.031
Delirium at admission	2343 (8.5)	822 (7.0)	−0.056
Length of stay, mean (SD), d	5.70 (6.69)	5.74 (6.97)	0.001

Abbreviations: eNICE, electronic National Institute for Health and Care Excellence; NA, not applicable; NICE, National Institute for Health and Care Excellence.

SI conversion factors: to convert albumin to grams per liter, multiply by 10; bilirubin to micromoles per liter, multiply by 17.104; creatinine to micromoles per liter, multiply by 88.4; glucose to millimoles per liter, multiply by 0.0555; hematocrit to a proportion of 1, multiply by 0.01; urea nitrogen to millimoles per liter, multiply by 0.357; and white blood cell count to ×10^9^/L, multiply by 0.001.

^a^ Data are presented as number (percentage) of patients unless otherwise indicated.

**Figure 1.**
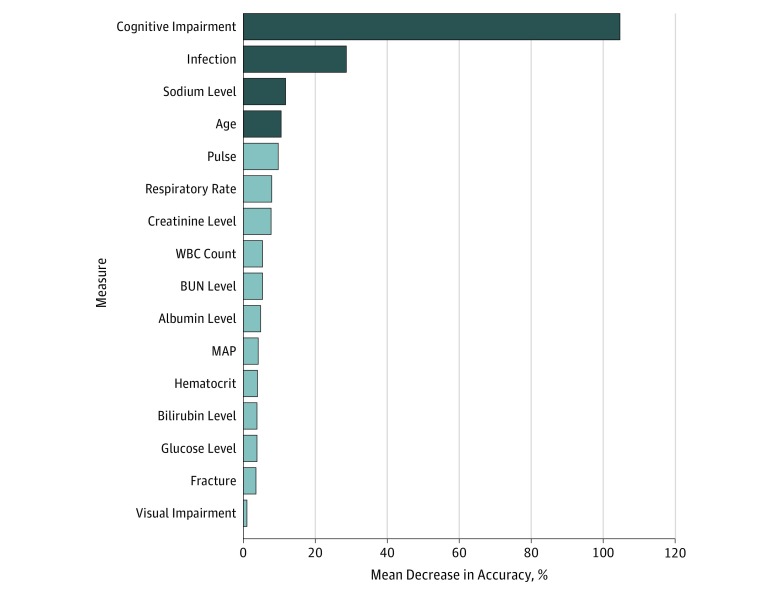
Importance Plot for the Random Forest Model Used to Generate the Consolidated National Institute for Health and Clinical Excellence (NICE) Score The mean decrease in accuracy shows the predictive power of each measure. Higher values indicate measures of higher importance. Cognitive impairment, infection, sodium level, and age were measures selected to be used in the consolidated NICE score. BUN indicates blood urea nitrogen; MAP, mean arterial pressure; WBC, white blood cell.

**Figure 2.**
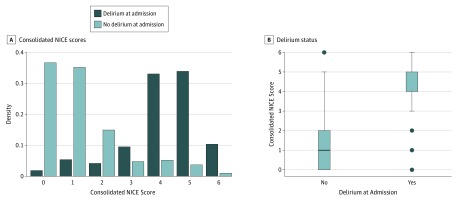
Comparison of the Consolidated National Institute for Health and Clinical Excellence (NICE) Score and Delirium Status A, The density histograms provide a probability breakdown of the consolidated NICE score by delirium status. B, The box plots show the association between the consolidated NICE score and delirium status in the derivation cohort. Center line in box indicates median; lower box border, 25th percentile or quartile 1; upper box border, 75th percentile or quartile 3; lower whisker, quartile 1 – 1.5 × interquartile range; upper whisker, quartile 3 + 1.5 × interquartile range.

In terms of predictive power and accuracy, cognitive impairment was the most important factor, followed by infection, sodium level, and age. The mean decrease in accuracies for the NICE features included in our random forest model is depicted in Figure 1. A cut point for age was determined using the median age node cutoff (≥80 years of age). A cut point for sodium level was determined using the median node cutoff and knowledge of normal sodium levels in VA patients. When we examined the breakdown of the sodium level cut point of 137 mEq/L (to convert to millimoles per liter, multiply by 1) (the median cut point determined by the random forest algorithm), no difference was found in the incidence of delirium at admission. Because the random forest model cannot pick a double cutoff, it picked within the reference range as the singular cutoff. At the VA, the reference range for sodium level in veterans is 135 to 145 mEq/L. Using our knowledge of VA patients, we decided on a double cutoff of less than 135 mEq/L or greater than 145 mEq/L as 1 point in our consolidated NICE score. eTable 1 in the Supplement gives the power of our 4 chosen predictive factors. There was an increase in the proportion of patients with delirium vs those without (eTable 1 in the Supplement). For the identified risk factors, patients with delirium were statistically more likely to be 80 years or older (1432 [14.4%] vs 911 [5.2%], *P* < .001), have cognitive impairment (2069 [41.8%] vs 274 [1.2%], *P* < .001), have infection diagnoses (1528 [16.6%] vs 815 [4.4%], *P* < .001), and have abnormal (<135 or >145 mEq/L) sodium levels (592 [11.0%] vs 1720 [8.0%], *P* < .001). Weights for the 4 delirium risk factors of cognitive impairment (3 points), age of 80 years or older (1 point), sodium level (1 point), and infection (1 point) were chosen based on mean decrease in accuracy measures (Table 2). A key observation was the reduced overlap of populations in the consolidated NICE model and the large difference in median consolidated NICE scores among those with delirium (Figure 2). The consolidated NICE scores discriminated between those with and without prevalent delirium (low risk, 252 [1.1%]; intermediate risk, 990 [29.4%]; high risk, 1101 [50.9%]; AUROC curve, 0.91; 95% CI, 0.90-0.92; *P* < .001) (eTable 2 in the Supplement). A comparison of the ROC curves of our consolidated NICE score vs those for the eNICE and Pendlebury NICE scores revealed the improvement in discrimination of the random forest prediction rule over existing approaches (eFigure in the Supplement).

**Table 2.** Independent Risk Factors for Delirium From NICE-Based Prediction Rules

Risk Factor for Delirium	NICE Meta-analysis^21^ Odds Ratio (95% CI)	Prediction Rule Weights		
		eNICE	Pendlebury NICE	Consolidated NICE
Cognitive impairment	6.3 (2.9-13.7)	4	2	3
Age ≥65 y	3.0 (1.2-7.7)	2	NA	NA
Age ≥80 y	5.2 (2.6-10.4)	3	2	1
Infection	3.0 (1.4-6.1)	2	1	1
Fracture	6.6 (2.2-19.3)	4	NA	NA
Visual impairment	1.7 (1.0-2.8)	1	1	NA
Severe illness	3.5 (1.5-8.2)	NA	NA	NA
Acute physiology score	NA	2	NA	NA
SIRS criteria^a^	NA	NA	1	NA
Serum sodium level	NA	NA	NA	1

Abbreviations: eNICE, electronic National Institute for Health and Care Excellence; NA, not applicable; NICE, National Institute for Health and Care Excellence; SIRS, systematic inflammatory response syndrome.

^a^ SIRS was noted as positive if at least 2 of the following were present: heart rate greater than 90 beats/min, respiratory rate greater than 20 breaths/min, and white blood cell count less than 4000/μL or greater than 12 000/μL (to convert to ×10^9^/L, multiply by 0.001). Temperature was not available in our data sets and therefore was excluded from SIRS calculations.

For the confirmation cohort, each of the 3 NICE rules were associated with delirium at admission (Table 3). Table 3 highlights the discriminatory function of each of the models. In each model, increasing delirium risk points was associated with increased risk of delirium. eTable 3 in the Supplement presents additional statistics that demonstrate the consolidated NICE score’s improvement in the percentage of those classified correctly and positive predictive value. All scores had high negative predictive values: 0.96 (consolidated NICE score), 0.98 (eNICE score), and 0.96 (Pendlebury NICE score). The consolidated NICE model had significantly higher predictive capability compared with the other models in both cohorts (eTable 3 in the Supplement). In the derivation cohort, the consolidated NICE score correctly classified 25 332 patients (91.7%) with an AUROC curve of 0.91 (95% CI, 0.91-0.92; *P* < .001), eNICE correctly classified 18 896 patients (68.4%) with an AUROC curve of 0.81 (95% CI, 0.80-0.82; *P* < .001), and the Pendlebury NICE score correctly classified 24 697 patients (89.4%) with an AUROC curve of 0.87 (95% CI, 0.86-0.88; *P* < .001). In the confirmation cohort, the consolidated NICE score correctly classified 10 882 patients (92.6%) with an AUROC curve of 0.91 (95% CI, 0.90-0.92; *P* < .001), eNICE correctly classified 8332 patients (70.9%) with an AUROC curve of 0.83 (95% CI, 0.81-0.84; *P* < .001), and the Pendlebury NICE score correctly classified 10 647 patients (90.6%) with an AUROC curve of 0.87 (95% CI, 0.86-0.88; *P* < .001).

**Table 3.** Comparison of 3 NICE Scores and Delirium Risk in the Derivation and Confirmation Cohorts

Validated Prediction Rule and Delirium Risk (Points)	Derivation		Confirmation	
	No. (%) With Delirium (n = 27 625)	AUROC Curve (95% CI)	No. (%) With Delirium (n = 11 752)	AUROC Curve (95% CI)
eNICE score				
Low (0-2)	75 (1.4)	0.81 (0.80-0.82)	27 (1.0)	0.83 (0.81-0.84)
Intermediate (3-5)	415 (3.5)		136 (2.6)	
High (6-9)	917 (11.9)		344 (11.3)	
Very high (10-18)	936 (38.9)		315 (36.0)	
Pendlebury NICE score				
Low (0-1)	71 (0.6)	0.87 (0.86-0.88)	30 (0.6)	0.87 (0.86-0.88)
Intermediate (2-4)	940 (7.0)		378 (6.8)	
High (5-7)	1332 (41.0)		414 (37.2)	
Consolidated NICE score				
Low (0-2)	252 (1.1)	0.91 (0.91-0.92)	103 (1.1)	0.91 (0.90-0.92)
Intermediate (3-4)	990 (29.4)		371 (27.7)	
High (5-6)	1101 (50.9)		348 (46.5)	

Abbreviations: AUROC, area under the receiver operating characteristic; eNICE, electronic National Institute for Health and Care Excellence; NICE, National Institute for Health and Care Excellence.

## Discussion

This analysis may provide an improvement over existing approaches to screening for delirium at admission by targeting 4 criteria and improving the accuracy compared with other algorithms. Furthermore, it revealed consistency in the NICE factors for delirium found in prior studies.^16,17,21^ By using the random forest method, we were able to identify cognitive impairment, age, sodium level, and infection as the variables that were primarily associated with delirium at admission. In addition, the use of separate but not prospective cohorts for derivation of the random forest algorithm and confirmation adds to the utility of this consolidated NICE delirium prediction rule for future research and implementation. This consolidated prediction rule may have clinical utility when used to identify patients in need of additional cognitive assessment and monitoring.

There may be some clinical advantages to recognizing delirium at admission. First, recent work has identified that a large percentage of patients at high risk for delirium may be missed with usual clinical care in the emergency and acute care settings.^8,9,10^ Of importance, patients with missed delirium have had negative outcomes in prior studies.^11,12,13^ Delirium at admission accounts for up to one-third of cases of delirium.^28^ Second, prior work with prevention strategies for delirium has excluded persons with delirium before enrollment.^29,30,31^ Third, the clinical guidelines lean toward routine screening for delirium specifically to identify and treat delirium early.^20,32,33^ Because of the low delirium recognition and poor outcomes associated with unrecognized delirium, an EMR-based delirium prediction tool may have some advantages after it is externally confirmed.

The value of any delirium prediction rule lies in its application to clinical practice. Because many cases of delirium are missed during routine clinical care, the use of an electronic tool to focus clinical assessment may efficiently direct clinical efforts. For example, if the consolidated NICE rule is embedded in the EMR and an electronic flag identifies the patient as high risk, a trained, frontline nurse can perform an ultrabrief assessment of cognitive function, such as the modified Richmond Agitation and Sedation Scale (15 seconds) or Months of the Year Backward (2 minutes),^34,35^ and initiate nonpharmacologic prevention strategies or ask for a more comprehensive assessment of delirium diagnosis and treatable causes. This brief, 2-step process may aid in quickly identifying patients at high risk for delirium and in need of more in-depth assessment and intervention for delirium. In addition, high delirium risk conveys prognostic information that is valuable to practitioners. For example, the eNICE confirmation study^16^ found that those at high and very high delirium risk at admission had increased length of stay, discharge to a rehabilitation facility, and readmissions compared with low-risk patients. As a result, systematic use of a delirium prediction rule, particularly an electronic measure, may efficiently identify patients who would benefit from additional clinical assessment.

### Strengths and Limitations

The strengths of this analysis are the use of large derivation and confirmation cohorts from centers across the United States, the random forest method, and the association with delirium at admission. Data were systematically collected from the VA EPRP and have been demonstrated to be highly reliable. Use of the NICE meta-analysis as the core of the random forest method enhances the validity and generalizability.

Despite the strengths of this work, there are significant limitations, and the results must be interpreted within these limits. First, generalizability is affected by the use of VA medical centers, which have a high proportion of men. Second, the VA system comprises 150 medical centers, and there is inherent variability in the assessment, diagnosis, and treatment of delirium. In this study, there was no external confirmation; additional, prospective confirmation in other health care systems, particularly those with EMR systems, is needed. Next, the EMR-based abstraction could limit the selection of additional variables because of availability, coding, or delay to diagnosis. Because our analysis was restricted to variables collected, there could be variables associated with delirium (eg, medications, prior living arrangement) that were not included in our random forest method. This factor may have affected the features selected by building random forests because of unavailability of some patient characteristics, such as vision or hearing data. Another limitation is the use of retrospective cohort EMR terms for delirium, which underestimates the prevalence of delirium.^36^ These terms also favor hyperactive delirium compared with hypoactive delirium, which is an inherent bias. Another limitation is that admissions were limited to 4 admission groups, which did not include groups at high risk for delirium, such as those with fractures.

The next step to move the consolidated NICE algorithm forward is confirmation in an external, prospective cohort with standardized assessment to improve validity for delirium at admission and delirium that develops after admission. Similar findings in such a prospective cohort with the reference standard delirium diagnosis may suggest that the internal validity of this retrospective study was not compromised.

## Conclusions

Practitioners frequently miss delirium at admission. Using advanced random forest methods for variable selection, this analysis found good discriminatory function for delirium at admission with 4 elements: cognitive impairment, age, sodium level, and infection. Further prospective examination of the consolidated NICE screening algorithm is required. Building screening algorithms such as this one into an EMR system in the future may help alert practitioners to individuals who would benefit from standardized cognitive assessment and appropriate interventions.

## Notes

Supplement. **eAppendix.** Methods of Creating the Consolidated National Institute for Health and Care Excellence **eTable 1.** Random Forest Selected Variables and Association With Delirium at Admission **eTable 2.** Categories of Consolidated NICE Delirium Prediction Rule **eTable 3.** Additional Performance Metrics of the 3 Prediction Rules **eFigure.** Receiver Operating Curves for the 3 NICE Rules for Delirium Upon Admission

## References

[1] American Psychiatric Association Diagnostic and Statistical Manual of Mental Disorders, Fifth Edition. Washington, DC: American Psychiatric Association; 2013.

[2] Han JH, Brummel NE, Chandrasekhar R, Exploring delirium’s heterogeneity: association between arousal subtypes at initial presentation and 6-month mortality in older emergency department patients. Am J Geriatr Psychiatry. 2016;25(3):-. doi:10.1016/j.jagp.2016.05.016 27623552 PMC5321606

[3] Jackson TA, MacLullich AMJ, Gladman JRF, Lord JM, Sheehan B Diagnostic test accuracy of informant-based tools to diagnose dementia in older hospital patients with delirium: a prospective cohort study. Age Ageing. 2016;45(4):505-511. doi:10.1093/ageing/afw065 27076526

[4] Collins N, Blanchard MR, Tookman A, Sampson EL Detection of delirium in the acute hospital. Age Ageing. 2010;39(1):131-135. doi:10.1093/ageing/afp201 19917632

[5] Lin RY, Heacock LC, Bhargave GA, Fogel JF Clinical associations of delirium in hospitalized adult patients and the role of on admission presentation. Int J Geriatr Psychiatry. 2010;25(10):1022-1029. doi:10.1002/gps.2500 20661879

[6] Timmons S, Manning E, Barrett A, Dementia in older people admitted to hospital: a regional multi-hospital observational study of prevalence, associations and case recognition. Age Ageing. 2015;44(6):993-999. doi:10.1093/ageing/afv131 26420638 PMC4621233

[7] Francis J, Kapoor WN Delirium in hospitalized elderly. J Gen Intern Med. 1990;5(1):65-79. doi:10.1007/BF02602312 2405116

[8] Clegg A, Westby M, Young JB Under-reporting of delirium in the NHS. Age Ageing. 2011;40(2):283-286. doi:10.1093/ageing/afq157 21169280

[9] Han JH, Zimmerman EE, Cutler N, Delirium in older emergency department patients: recognition, risk factors, and psychomotor subtypes. Acad Emerg Med. 2009;16(3):193-200. doi:10.1111/j.1553-2712.2008.00339.x 19154565 PMC5015887

[10] Rice KL, Bennett MJ, Clesi T, Linville L Mixed-methods approach to understanding nurses’ clinical reasoning in recognizing delirium in hospitalized older adults. J Contin Educ Nurs. 2014;45(3):136-148. doi:10.3928/00220124-20140219-02 24527890

[11] Kakuma R, du Fort GG, Arsenault L, Delirium in older emergency department patients discharged home: effect on survival. J Am Geriatr Soc. 2003;51(4):443-450. doi:10.1046/j.1532-5415.2003.51151.x 12657062

[12] Cole M, McCusker J, Dendukuri N, Han L The prognostic significance of subsyndromal delirium in elderly medical inpatients. J Am Geriatr Soc. 2003;51(6):754-760. doi:10.1046/j.1365-2389.2003.51255.x 12757560

[13] Bellelli G, Nobili A, Annoni G, ; REPOSI (REgistro POliterapie SIMI) Investigators Under-detection of delirium and impact of neurocognitive deficits on in-hospital mortality among acute geriatric and medical wards. Eur J Intern Med. 2015;26(9):696-704. doi:10.1016/j.ejim.2015.08.006 26333532

[14] Inouye SK Delirium in older persons. N Engl J Med. 2006;354(11):1157-1165. doi:10.1056/NEJMra052321 16540616

[15] Teodorczuk A, Reynish E, Milisen K Improving recognition of delirium in clinical practice: a call for action. BMC Geriatr. 2012;12(1):55. doi:10.1186/1471-2318-12-55 22974329 PMC3463439

[16] Rudolph JL, Doherty K, Kelly B, Driver JA, Archambault E Validation of a delirium risk assessment using electronic medical record information. J Am Med Dir Assoc. 2016;17(3):244-248. doi:10.1016/j.jamda.2015.10.020 26705000

[17] Pendlebury ST, Lovett N, Smith SC, Cornish E, Mehta Z, Rothwell PM Delirium risk stratification in consecutive unselected admissions to acute medicine: validation of externally derived risk scores. Age Ageing. 2016;45(1):60-65. doi:10.1093/ageing/afv177 26764396 PMC4711661

[18] Marcantonio ER, Goldman L, Mangione CM, A clinical prediction rule for delirium after elective noncardiac surgery. JAMA. 1994;271(2):134-139. doi:10.1001/jama.1994.03510260066030 8264068

[19] Inouye SK, Westendorp RGJ, Saczynski JS Delirium in elderly people. Lancet. 2014;383(9920):911-922. doi:10.1016/S0140-6736(13)60688-1 23992774 PMC4120864

[20] Young J, Murthy L, Westby M, Akunne A, O’Mahony R; Guideline Development Group Diagnosis, prevention, and management of delirium: summary of NICE guidance. BMJ. 2010;341:c3704. doi:10.1136/bmj.c3704 20667955

[21] National Institute for Health and Clinical Excellence DELIRIUM: Diagnosis, Prevention and Management. Clinical Guideline 103. London, England: National Institute for Health and Clinical Excellence; 2010.

[22] Goulet JL, Erdos J, Kancir S, Measuring performance directly using the Veterans Health Administration electronic medical record: a comparison with external peer review. Med Care. 2007;45(1):73-79. doi:10.1097/01.mlr.0000244510.09001.e5 17279023 PMC3460379

[23] Collins GS, Reitsma JB, Altman DG, Moons KG Transparent Reporting of a Multivariable Prediction Model for Individual Prognosis or Diagnosis (TRIPOD). Ann Intern Med. 2015;162(10):735-736. doi:10.7326/L15-5093-2 25984857

[24] Knaus WA, Wagner DP, Draper EA, The APACHE III prognostic system: risk prediction of hospital mortality for critically ill hospitalized adults. Chest. 1991;100(6):1619-1636. doi:10.1378/chest.100.6.1619 1959406

[25] Liaw A, Wiener M Classification and regression by randomforest. R News. 2002;2(3):18-22.

[26] Breiman L Manual: Setting Up, Using, and Understanding Random Forests V 4.0. http://www.stat.berkeley.edu/~breiman/Using_random_forests_v4.0.pdf. Accessed January 26, 2017.

[27] Breiman L, Cutler A, Liaw A, Wiener M Breiman and Cutler's Random Forests for Classification and Regression. 2015. https://cran.r-project.org/web/packages/randomForest/randomForest.pdf. Accessed January 26, 2017.

[28] Siddiqi N, House AO, Holmes JD Occurrence and outcome of delirium in medical in-patients: a systematic literature review. Age Ageing. 2006;35(4):350-364. doi:10.1093/ageing/afl005 16648149

[29] Teale E, Young J Multicomponent delirium prevention: not as effective as NICE suggest? Age Ageing. 2015;44(6):915-917. doi:10.1093/ageing/afv120 26316509

[30] Martinez F, Tobar C, Hill N Preventing delirium: should non-pharmacological, multicomponent interventions be used? a systematic review and meta-analysis of the literature. Age Ageing. 2015;44(2):196-204. doi:10.1093/ageing/afu173 25424450

[31] Hshieh TT, Yue J, Oh E, Effectiveness of multicomponent nonpharmacological delirium interventions: a meta-analysis. JAMA Intern Med. 2015;175(4):512-520. doi:10.1001/jamainternmed.2014.7779 25643002 PMC4388802

[32] Barr J, Fraser GL, Puntillo K, ; American College of Critical Care Medicine Clinical practice guidelines for the management of pain, agitation, and delirium in adult patients in the intensive care unit. Crit Care Med. 2013;41(1):263-306. doi:10.1097/CCM.0b013e3182783b72 23269131

[33] American Geriatrics Society Expert Panel on Postoperative Delirium in Older Adults American Geriatrics Society abstracted clinical practice guideline for postoperative delirium in older adults. J Am Geriatr Soc. 2015;63(1):142-150. doi:10.1111/jgs.13281 25495432 PMC5901697

[34] Chester JG, Beth Harrington M, Rudolph JL; VA Delirium Working Group Serial administration of a modified Richmond Agitation and Sedation Scale for delirium screening. J Hosp Med. 2012;7(5):450-453. doi:10.1002/jhm.1003 22173963 PMC4880479

[35] Yevchak AM, Doherty K, Archambault EG, Kelly B, Fonda JR, Rudolph JL The association between an ultrabrief cognitive screening in older adults and hospital outcomes. J Hosp Med. 2015;10(10):651-657. doi:10.1002/jhm.2450 26374602 PMC4594206

[36] Hope C, Estrada N, Weir C, Teng CC, Damal K, Sauer BC Documentation of delirium in the VA electronic health record. BMC Res Notes. 2014;7:208. doi:10.1186/1756-0500-7-208 24708799 PMC3985575

