# Supplementary material for: Performance of Electronic Prediction Rules for Prevalent Delirium at Hospital Admission
Source: JAMA Netw Open. 2018 Aug 24;1(4):e181405. doi: 10.1001/jamanetworkopen.2018.1405 (PMC6324279; doi:10.1001/jamanetworkopen.2018.1405)
Supplement: Supplement. — eAppendix. Methods of Creating the Consolidated National Institute for Health and Care Excellence eTable 1. Random Forest Selected Variables and Association With Delirium at Admission eTable 2. Categories of Consolidated NICE Delirium Prediction Rule eTable 3. Additional Performance Metrics of the 3 Prediction Rules eFigure. Receiver Operating Curves for the 3 NICE Rules for Delirium Upon Admission [file jamanetwopen-1-e181405-s001.pdf]

Supplementary Online Content

Halladay CW, Sillner AY, Rudolph JL. Performance of Electronic Prediction Rules for Prevalent Delirium at Hospital Admission. *JAMA Netw Open.* 2018;1(4):e181405. doi:10.1001/jamanetworkopen.2018.1405

eAppendix. Methods of Creating the Consolidated National Institute for Health and Care Excellence

eTable 1. Random Forest Selected Variables and Association With Delirium at Admission

eTable 2. Categories of Consolidated NICE Delirium Prediction Rule

eTable 3. Additional Performance Metrics of the 3 Prediction Rules

eFigure. Receiver Operating Curves for the 3 NICE Rules for Delirium Upon Admission

This supplementary material has been provided by the authors to give readers additional information about their work.

© 2018 Halladay CW et al. *JAMA Network Open.*

eAppendix. Methods of Creating the Consolidated National Institute for Health and Care Excellence are broken into the following four steps:

1) Random Forest Selection; (figure 2)
2) Confirmation of Findings; (eTable 1)
3) Assigning of Points or Weights; (Table 2)
4) Association with Delirium at Hospital Admission (eTable 2) and Additional Performance Characteristics of the prediction rules (eTable 3)

© 2018 Halladay CW et al. *JAMA Network Open.*

2) Confirmation of Findings was determined within the validation cohort, comparing those without and with delirium at hospital admission. Variables including within the Consolidated NICE Prediction Rule reach significance of < 0.001.

eTable 1. Random Forest Selected Variables and association with Delirium at Admission

Variable	Validation Cohort (n = 27625)	No Delirium at Admission (n = 25282)	Delirium at Admission (n = 2343)	p-value
**Age >= 80 years, n (%)**				p<.001
**No**	17672 (64.0)	16761 (94.8)	911 (5.2)	
**Yes**	9953 (36.0)	8521 (85.6)	1432 (14.4)	
**Cognitive Impairment, n (%)**				p<.001
**No**	22671 (82.1)	22397 (98.8)	274 (1.2)	
**Yes**	4954 (17.9)	2885 (58.2)	2069 (41.8)	
**Infection, n (%)**				p<.001
**No**	18445 (66.8)	17630 (95.6)	815 (4.4)	
**Yes**	9180 (33.2)	7652 (83.4)	1528 (16.6)	
**Sodium < 135 mg/dL or Sodium > 145 mg/dL, n (%)**				p<.001
**No**	21612 (80.1)	19892 (92.0)	1720 (8.0)	
**Yes**	5385 (19.9)	4793 (89.0)	592 (11.0)	

© 2018 Halladay CW et al. *JAMA Network Open.*

3) Assigning of Points or Weights was given to each of the Consolidated NICE variables. The highest weighted category was prior cognitive impairment with a weight of 3 points. Cognitive impairment is a known risk factor for delirium prediction risk and within the Consolidated NICE model is given the largest weight. Remaining variables were each given a weight of 1 point based on the Random Forest Selection modeling. (Table 2)

© 2018 Halladay CW et al. *JAMA Network Open.*

4) We then used the weighted Consolidate NICE Prediction Score to categorize individuals within the Validation Cohort into three risk categories: Low, Intermediate, and High which ranged from 0 to 6 points. Individuals within the Low category obtained a score ranging from 0-2 points; Intermediate was 3-4 points; and the Highest Risk Category was 5-6 points. Within the Validation Cohort, almost half of individuals with delirium were categorized as High Risk based on their Consolidated NICE score. Close to 30% were at Intermediate risk and approximately 1% were categorized as low risk using the Consolidated NICE score.

eTable 2. Categories of Consolidated NICE Delirium Prediction Rule

	**Score Range**	**Individuals with Delirium at Hospital Admission n (%)**
**Consolidated NICE score**	Low (0-2)	252 (1.1)
	Intermediate (3-4)	990 (29.4)
	High (5-6)	1101 (50.9)

eTable 3. Additional performance metrics of the 3 prediction rules

Prediction Rule and cut-off	Percent Classified Correctly	Positive Predictive Value	Negative Predictive Value
eNice >= 6	70.90%	17%	98%
NICE Pendlebury >= 5	90.60%	37%	96%
Consolidate NICE >= 5	92.60%	47%	96%

© 2018 Halladay CW et al. *JAMA Network Open.*

eFigure. Receiver Operating Curves for the 3 NICE Rules for delirium upon admission

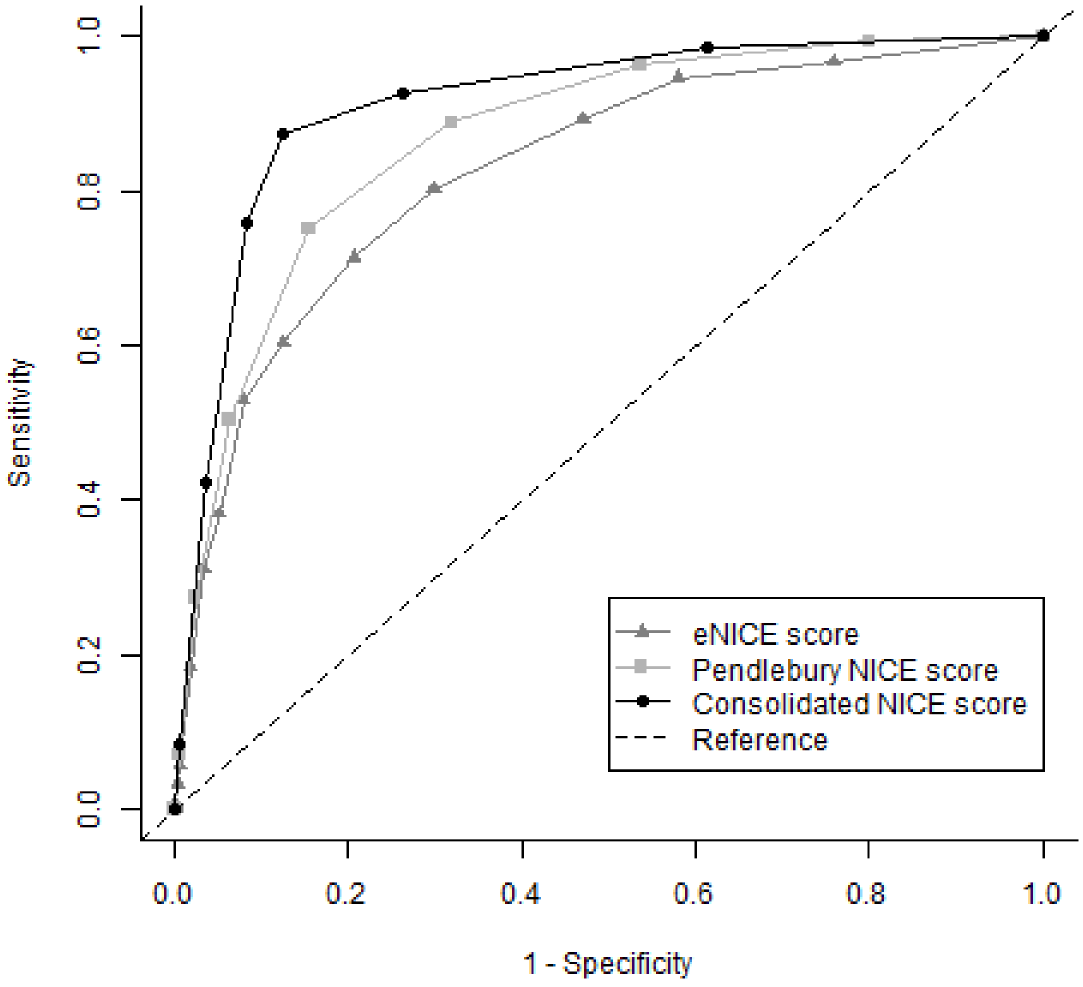

© 2018 Halladay CW et al. *JAMA Network Open.*
